# Editorial: Functionality and applications of phytochemicals in aquaculture nutrition

**DOI:** 10.3389/fvets.2023.1218542

**Published:** 2023-05-26

**Authors:** Hany M. R. Abdel-Latif, Sevdan Yilmaz, Dariusz Kucharczyk

**Affiliations:** ^1^Department of Poultry and Fish Diseases, Faculty of Veterinary Medicine, Alexandria University, Alexandria, Egypt; ^2^Department of Aquaculture, Faculty of Marine Sciences and Technology, Çanakkale Onsekiz Mart University, Çanakkale, Turkey; ^3^Department of Ichthyology and Aquaculture, University of Warmia and Mazury, Olsztyn, Poland; ^4^Department of Research and Development, Chemprof, Olsztyn, Poland

**Keywords:** phytochemicals, immunity, growth, gene expression, antioxidant status, resistance, gut health, microbiome

The aquaculture industry is a fast-growing sector amongst the food production sectors, as its growth is strongly associated with the continuous increase of the global population. This industry effectively contributes to food security by providing nutritive and healthful aquatic foods and manufactured products for human beings (1). The chief target of this valuable sector is to raise the production rates with cost-effective benefits to enlarge the profitability margins for fish farmers. This goal has directed the visions of farmers and aquaculturists for the application of intensive farming systems by increasing the total biomass per unit surface area or water volume. Although this farming system has maximized productivity, it may induce several drawbacks and side effects if the fish are not carefully monitored or controlled environments are not maintained. If these conditions are not found, deterioration of the water quality parameters (particularly elevated un-ionized ammonia, nitrite, nitrate, organic matter, or decreased dissolved oxygen levels) will occur. Furthermore, the possibility of flourishing emergent diseases (bacterial, fungal, or viral) will occur and spread rapidly among the farmed fish (2). Deteriorated water quality will induce stressful environmental conditions, disrupt the physiological responses of the exposed fish, trigger immune suppression, compromise health, and consequently cause high mortalities. Besides, the emergence of infectious pathogens will cause heavy kills and subsequent critical economic loss.

On the other hand, the application of antibiotics or antimicrobials to control bacterial diseases has been banned and prohibited in the aquaculture industry in various countries worldwide. It was noticed that the overuse or misuse of these chemicals had been reported to induce adverse impacts on human consumers due to the deposition of drug residues in the filets of the treated fish or shrimp (3). Besides, there is evidence of flourishing antibiotic-resistant strains, which will be more dangerous as they can resist treatment therapeutics (4) and possible environmental toxicity concerns (5). These compounds can give rise to extremely negative impacts on the natural biocenoses located near aquaculture facilities and cause the elaboration of bacterial resistance to the used antibiotics (6). For these reasons, efforts have been made by scientists and researchers to find economical, cost-effective, durable, non-toxic, and environmentally safe alternatives in order to limit antibiotic usage, enhance the health status, welfare, and overall performance, and reduce stress and mortalities of farmed fish and shrimp (7, 8).

Throughout the past years, plentiful research and review articles have been published and focused principally on the beneficial effects of herbal supplements or phytobiotics in aquaculture. The term “phytobiotics” can be defined as plants and their part(s), or extracts that can be used as feed additives with known efficacy owing to their health benefits and unique functions. In aquaculture, these ingredients are helpful in improving growth rate, immunity, redox state (balance between oxidants and antioxidants), and disease resistance of several fish and shrimp species (9–12). These effects are not only limited to the plant ingredients but also to their phytochemical compounds (13, 14) and the essential oils (EOs) extracted from these plants as secondary metabolites (15, 16).

Phytochemicals or “Phytonutrients” are beneficial chemicals that are present in plants and produce considerable protection of plants against a variety of fungi, bacteria, and viruses. They are important in herbal medicine and have been widely used in the last years in several Asian countries, especially in the diets of humans and also terrestrial animals. In human medicine, reports showed their potential efficacy for decreasing the risks of developing cancers, hypertension, diabetes, heart diseases, and several others. Their mode of action depends mainly on their antioxidant and immune-stimulant roles. They comprise an ample number of several plant-derived bioactive functional ingredients that are generally obtained from vegetables, fruits, cereals, and beans (17, 18).

They contain many essential molecules like flavonoids, alkaloids, polyphenols, isoflavonoids, phenolics, pigments, terpenoids, glucosinolates, carotenoids, and anthocyanins (13, 14, 17, 18). These molecules can be used in fish and shrimp diets as functional feed additives to enhance growth, support immunity, reduce stress, potentiate antioxidant capacity, and enhance disease resistance. Nutritionists have estimated and listed more than 4,000 phytochemicals; however, about 150 compounds from these compounds have been studied in depth for nutrition purposes. More research studies are still necessary in aquaculture nutrition to elucidate which phytochemicals may benefit the farmed aquatic organisms. Their modes of action should also be studied in detail. For these reasons, with this Research Topic, we aimed to spotlight the functionality and applicability of phytochemicals to enhance the welfare and performance of fish and shrimp species. This Topic was of vital significance not only to boost the health status and wellbeing of fish and shrimp but also to maintain aquaculture sustainability.

In general, reports showed that phytochemicals have essential functions when being used in aquafeed to enhance immunity and disease resistance (17). They can also be used as growth promoters, endocrine modulators (18), and antistress agents (19). These ingredients can be used as an effective antimicrobial approach in aquaculture against a variety of fish pathogenic agents (20). With a particular concern, reports showed that phenolic compounds possess antibacterial, antifungal, antiviral, and antiprotozoal activities against bacterial, fungal, viral, and protozoal agents that affect fish, as reviewed by Beltrán and Esteban (21). Hence, they can be helpful in combating challenging infectious agents with a possible application as antibiotic alternatives (10). Despite the above-mentioned health benefits and functions of using phytochemicals in aquafeed, a detailed comprehensive review regarding their residual effects in fish and shrimp tissues must be studied to ensure their safety for human consumption. Their toxicity levels should also be determined and monitored accurately before their incorporation into fish and shrimp diets to determine their optimal dietary supplementation levels to avoid negative impacts on the treated organisms. Moreover, harmonized research efforts are still required to determine the mechanisms of action of these ingredients inside the fish and shrimp bodies.

This Research Topic contained a variety of research aspects that associated with the possibilities and/or potential application of phytochemicals in aquafeed and the assessment of their effects on growth, immune responses, redox status, gut microbiome, and intestinal histomorphology of finfish and shrimp species. The beneficial roles of these phytochemicals in (a) reducing the negative impacts of stressors, (b) enhancing disease resistance against challenging pathogens, and (c) promoting the welfare and wellbeing of farmed fish and shrimp species are also described. Nevertheless, further detailed research is still necessary to better understand the mechanisms and/or the modes of action of these beneficial phytochemicals for improving the overall performances of farmed fish and shrimp.

Due to the importance of this subject in the field of aquaculture, our team was concerned with presenting a Research Topic in Frontiers in Veterinary Science (section Animal Nutrition and Metabolism) to direct the attention of researchers to this issue to gather relevant research papers and investigations on this issue. We tried to concentrate on a variety of points of view in the field of running the Research Topic. In this context, we succeeded in collecting and publishing five papers from 33 authors from around the world. The papers collected on this Research Topic signify the recent data and information associated with using phytochemicals in aquaculture studies.

Liu et al. presented a paper relating to the importance of the phytochemical properties of several dietary doses of non-starch polysaccharides (NSPs) derived from the plant cell walls on digestibility, intestinal digestive enzyme activities, growth, and intestinal histomorphology of a carnivorous fish species known as largemouth bass (*Micropterus salmoides*) juveniles. After an 8-week feeding experiment, those authors found that higher levels of dietary NSPs adversely affect the intestinal digestive enzyme activities and intestinal histomorphology of juvenile largemouth bass. These findings are associated with reducing the apparent nutrient digestibility and the growth performance of this fish species. In this study, Liu et al. suggested that juvenile largemouth bass, as carnivorous fish, has a limited tolerability to dietary NSPs with a maximum not exceeding 5.51%.

Martínez-Antequera et al. presented an *in vitro* approach to assess the biological roles of phenolic compounds obtained from two types of wine by-products (wine bagasse and wine lees) on the digestive physiology (particularly in terms of the digestive bioaccessibility and the digestive proteases) of two fish species with different feeding habits: an omnivorous fish such as gilthead sea bream (*Sparus aurata*) and an herbivorous fish such as flathead gray mullet (*Mugil cephalus*). Those authors analyzed 13 phenolic compounds in the tested wine product. Moreover, they noticed great variations in the release patterns of phenolic compounds with time. This notice suggests an important effect of gut transit rates on the net bioavailability of a given phenolic compound in live fish. According to this finding, they proposed to conduct an *in vivo* experiment in the fish species to validate the obtained *in vitro* results to stand over solid information for potential application in fish diets.

Yousefi et al. evaluated the dietary effects of different hyssop (*Hyssopus officinalis*) extract levels on the physiological responses and antioxidant activities of juvenile rainbow trout (*Oncorhynchus mykiss*) subjected to thermal stress. Those authors investigated the functional bioactive ingredients in *H. officinalis* extract using GC-MS analysis. Yousefi et al. observed that rainbow trout exposed to thermal stress showed significant increases in the plasma stress biomarkers (including glucose, cortisol, LDH, and lactate) and the oxidative stress biomarkers in the gill tissues (including glutathione peroxidase, glutathione reductase, and glutathione-S-transferase enzyme activities). After a 70-day feeding trial, those authors reported that dietary supplementation with *H. officinalis* extract (particularly at 250 mg/kg) significantly ameliorated the aforementioned parameters.

## Author contributions

HA-L, SY, and DK have contributed to writing this editorial. All authors contributed equally to the article and approved the submitted version.

## References

[1] FAO. The State of World Fisheries and Aquaculture. Opportunities, and Challenges. Rome: Food and Agriculture Organization of the United Nations (2021).

[2] Zaki MA Khalil HS Allam BW Khalil RH Basuini MFE Nour AEAM. Assessment of zootechnical parameters, intestinal digestive enzymes, haemato-immune responses, and hepatic antioxidant status of *Pangasianodon hypophthalmus* fingerlings reared under different stocking densities. Aquacult. Int. (2023). 10.1007/s10499-023-01092-w

[3] Cabello FC. Heavy use of prophylactic antibiotics in aquaculture: a growing problem for human and animal health and for the environment. Environ Microbiol. (2006) 8:1137–44. 10.1111/j.1462-2920.2006.01054.x 16817922

[4] Alderman DJ Hastings TS. Antibiotic use in aquaculture: development of antibiotic resistance – potential for consumer health risks. Int J Food Sci Technol. (1998) 33:139–55. 10.1046/j.1365-2621.1998.3320139.x

[5] Holmström K Gräslund S Wahlström A Poungshompoo S Bengtsson B-E Kautsky N. Antibiotic use in shrimp farming and implications for environmental impacts and human health. Int J Food Sci Technol. (2003) 38:255–66. 10.1046/j.1365-2621.2003.00671.x

[6] Feng Y Lu Y Chen Y Xu J Jiang J. Microbial community structure and antibiotic resistance profiles in sediments with long-term aquaculture history. J Environ Managt. (2023) 341:118052. 10.1016/j.jenvman.2023.118052 37141714

[7] Abdel-Latif HMR Dawood MAO Alagawany M Faggio C Nowosad J Kucharczyk D. Health benefits and potential applications of fucoidan (FCD) extracted from brown seaweeds in aquaculture: an updated review. Fish Shellfish Immunol. (2022) 122:115–30. 10.1016/j.fsi.2022.01.039 35093524

[8] Nowosad J Jasiński S Arciuch-Rutkowska M Abdel-Latif HMR Wróbel M Mikiewicz M. Effects of bee pollen on growth performance, intestinal microbiota and histomorphometry in African catfish. Animals. (2023) 13:132. 10.3390/ani13010132 36611741 PMC9817710

[9] Abdel-Latif HMR Abdel-Daim MM Shukry M Nowosad J Kucharczyk D. Benefits and applications of *Moringa oleifera* as a plant protein source in aquafeed: a review. Aquaculture. (2022) 547:737369. 10.1016/j.aquaculture.2021.737369

[10] Li M Wei D Huang S Huang L Xu F Yu Q. Medicinal herbs and phytochemicals to combat pathogens in aquaculture. Aquac Int. (2022) 30:1239–59. 10.1007/s10499-022-00841-7

[11] Abd-Elaziz RA Shukry M Abdel-Latif HMR Saleh RM. Growth-promoting and immunostimulatory effects of phytobiotics as dietary supplements for *Pangasianodon hypophthalmus* fingerlings. Fish Shellfish Immunol. (2023) 133:108531. 10.1016/j.fsi.2023.108531 36639065

[12] Yilmaz S Sanver Çelik E Ergün S Ahmadifar E Abdel-Latif HMR. Effects of dietary walnut (*Juglans regia*) leaves extract on immunity, gene expression responses, and disease resistance in *Oreochromis niloticus*. Fish Shellfish Immunol. (2023) 135:108656. 10.1016/j.fsi.2023.108656 36868534

[13] Ahmadifar E Yousefi M Karimi M Fadaei Raieni R Dadar M Yilmaz S. Benefits of dietary polyphenols and polyphenol-rich additives to aquatic animal health: an overview. Rev Fish Sci Aquac. (2021) 29:478–511. 10.1080/23308249.2020.1818689

[14] Naiel MAE El-Kholy AI Negm SS Ghazanfar S Shukry M Zhang Z. A mini-review on plant-derived phenolic compounds with particular emphasis on their possible applications and beneficial uses in aquaculture. Ann. Anim. Sci. (2023). 10.2478/aoas-2023-0007

[15] Dawood MAO El Basuini MF Zaineldin AI Yilmaz S Hasan MT Ahmadifar E. Antiparasitic and antibacterial functionality of essential oils: An alternative approach for sustainable aquaculture. Pathogens. (2021) 10:185. 10.3390/pathogens10020185 33572193 PMC7914417

[16] Dawood MAO El Basuini MF Yilmaz S Abdel-Latif HMR Alagawany M Kari ZA. Exploring the roles of dietary herbal essential oils in aquaculture: a review. Animals. (2022) 12:823. 10.3390/ani12070823 35405814 PMC8996993

[17] Chakraborty SB Hancz C. Application of phytochemicals as immunostimulant, antipathogenic and antistress agents in finfish culture. Rev Aquac. (2011) 3:103–19. 10.1111/j.1753-5131.2011.01048.x

[18] Chakraborty SB Horn P Hancz C. Application of phytochemicals as growth-promoters and endocrine modulators in fish culture. Rev Aquac. (2014) 6:1–19. 10.1111/raq.12021

[19] Taştan Y Salem MOA. Use of phytochemicals as feed supplements in aquaculture: a review on their effects on growth, immune response, and antioxidant status of finfish. J Agric Prod. (2021) 2:32–43. 10.29329/agripro.2021.344.5

[20] Nik Mohamad Nek Rahimi N Natrah I Loh J-Y Ervin Ranzil FK Gina M Lim S-HE. Phytocompounds as an alternative antimicrobial approach in aquaculture. Antibiotics. (2022) 11:469. 10.3390/antibiotics11040469 35453220 PMC9031819

[21] Beltrán JMG Esteban MÁ. Nature-identical compounds as feed additives in aquaculture. Fish Shellfish Immunol?. (2022) 123:409–16. 10.1016/j.fsi.2022.03.010 35331881

